# Hematological Adverse events and Sustained Viral Response in Children Undergoing Therapy for Chronic Hepatitis C Infection

**DOI:** 10.5812/kowsar.1735143X.789

**Published:** 2011-12-20

**Authors:** Malgorzata Pawlowska, Malgorzata Pilarczyk, Anna Foksinska, Ewa Smukalska, Waldemar Halota

**Affiliations:** 1 Department of Infectious Diseases and Hepatology, Collegium Medicum, n.Copernicus University, Bydgoszcz, poland

**Keywords:** Hepatitis C, Child, Therapeutics

## Abstract

**Background:**

Treatment of hepatitis C virus (HCV) infection with interferon (IFN) and ribavirin (RBV) is associated with adverse events, which may affect the patient's adherence to the treatment regimen and the treatment efficacy.

**Objectives:**

The aim of this study was to assess the sustained viral response (SVR) and interdependence between the haematological characteristics (leukocyte count, platelet count, and haemoglobin levels) in patients with chronic hepatitis C (CHC) infection during treatment with IFN and RBV.

**Patients and Methods:**

We conducted a retrospective cohort study of 170 children with CHC infection who completed treatment with IFN-α and RBV. The children were divided into 2 groups: the first group (group I, n = 119) underwent a 48-week course of treatment with recombinant IFN α-2b (Intron A) at a dosage of 3 MU 3 times a week subcutaneously and RBV at a dosage of 15 mg/kg per day orally, and the second group (group II, n = 51) was administered pegylated IFN (peg-IFN)-α-2b (PegIntron) at a dosage of 1.5 μg/kg per week subcutaneously and RBV at a dosage of 15 mg/kg per day orally for 48 weeks. The dose of IFN was not adjusted but that of ribavirin was in 2 children from group II. Hematological growth factors and erythropoietin were not used. SVR was defined as undetectable serum HCV RNA 24 weeks after the end of treatment (study week 72). Serum HCV RNA was determined by performing polymerase chain reaction, and the HCV genotypes and hematological parameters were evaluated. Serum HCV RNA levels were analysed by descriptive statistics. Means and standard deviations were calculated for values collected at the baseline, on the 12th and 48th weeks during treatment, and after 24 weeks of untreated follow-up (study week 72).

**Results:**

Eighty-six (50%) of the 170 patients who underwent treatment achieved SVR: 62 (51%) out of 119 children from group I and 24 (47%) out of 51 from group II. The mean serum hemoglobin levels and leukocyte and platelet counts at week 12 were significantly lower than the baseline values in both responders and non-responders from both the groups (P < 0.05). In the responders in group I, the mean levels of serum hemoglobin after 24 weeks of treatment and at the end of therapy were significantly lower than the mean levels at baseline. In the group treated with peg-IFN-α-2b and RBV (group II), the mean serum hemoglobin levels at week 12 was lower in the responders than in the non-responders (P < 0.05). The decrease in the hemoglobin levels was associated with viral response. In both the responders and non-responders from both the groups, leukocyte counts decreased during treatment, and after 12 weeks, they were more significantly lower than the baseline value. The decrease was more marked in children treated with peg-IFN-α-2b + RBV (P < 0.05). After 12 weeks of treatment, the platelet count was low in children from group II who had achieved SVR.

**Conclusions:**

A mild decrease in hemoglobin levels and leukocyte and platelet counts during treatment with IFN and RBV in children with CHC infection may be factors responsible for SVR induction.

## 1. Background

Hepatitis C virus (HCV) infection is the leading cause of chronic liver disease and cirrhosis, and it is the most common underlying diagnosis in patients who undergo liver transplant. Children form only a small proportion of the HCV-infected population, but there are a significant number of children with chronic HCV (CHC) infection [1]. In children, HCV infection is considered to be a mild asymptomatic disease that has a limited histological spectrum and in which the serum alanine aminotransferase (ALT) levels are mostly normal. Clinical symptoms of HCV infection are observed in approximately 20% of the infected children, with hepatomegaly being the most frequently observed sign [2]. Because of the possibility of serious late clinical consequences such as liver cirrhosis and hepatocellular carcinoma, children with CHC should be administered antiviral treatment [3]. The main goal of therapy for HCV infection is to achieve a sustained virological response (SVR), which is practically equivalent to the eradication of HCV infection and cure of the underlying HCV-induced liver disease. Currently, the standard treatment for adults with HCV infection is a combination therapy with pegylated interferon (peg-IFN)-α-2b and ribavirin (RBV). The application of this therapy in children was approved by the Food and Drug Administration in December 2008 and by the European Agency for the Evaluation of Medicinal Products in November 2009. This therapy is very effective and secures a high quality of life for the patient, which is very important, especially in the case of children [2][4][5].

The efficacy of the combination therapy with peg-IFN-α-2b and RBV in children with HCV infection is comparable to and even higher than that reported in adults. This possibly better viral response in children may be associated with shorter duration of HCV infection, lower frequency of liver fibrosis and iron overload, and lower rate of risky behavior (intravenous drug usage, alcohol consumption, etc.) and comorbidities [4][5][6]. Treatment with peg-IFNα-2b and RBV is associated with several, and sometimes severe, adverse events, which may affect the treatment efficacy and the patient's adherence to the treatment regimen. Side effects of such a combination therapy mainly include fever, headache, fatigue, flu-like syndrome, and hematological disorders such as leucopenia, thrombocytopenia, and anemia. Depression, weight loss, thyroiditis, and other autoimmune diseases such as decrease in height growth, especially in children, have also been reported to be the side effects of the combination therapy [5][7][8].

## 2. Objectives

The aim of this study was to assess the SVR and interdependence between the hematological parameters (leukocyte count, platelet count, hemoglobin levels) during the treatment of CHC infection with IFN and RBV.

## 3. Patients and Methods

We conducted a retrospective cohort study of 170 children with CHC infection who completed treatment with recombinant IFN-α-2b, peg-IFN-α-2b, and RBV (Schering-Plough) from March 2000 to June 2006. The protocol was approved by the ethics committee of the Collegium Medicum of the Nicolaus Copernicus University, Bydgoszcz, Poland. Informed consent was obtained in writing from the legal guardian of each patient before the treatment was initiated. Children who were considered eligible for the treatment had CHC infection that had been diagnosed on the basis of the presence of HCV RNA in the serum and the histopathological changes in the liver. During the treatment, patients returned to the clinic at regular intervals, at which time their blood samples were collected for determining serum HCV RNA viral load and ALT activity. A complete blood count was performed during each clinical visit. Baseline characteristics of the examined children have been listed in Table 1.

**Table 1:** Baseline Characteristics of the Patients (n = 170)

	**Treated With IFN a α-2b + RBV a, Group I (n = 119)**	**Treated With Pegylated IFN -α-2b + RBV, Group II (n = 51)**
Sex		
Female	41	15
Male	78	36
Age, y, (mean)	1–18 (11.7)	8–18 (13.6)
HCV genotype, No. (%)		
1	48/75 (64)	27/51 (53)
4	27/75 (36)	24/51 (47)
Baseline HCV RNA, IU/mL ^b^, mean ± SD	3.58 ± 1.17 × 105	8.98 ± 6.19 × 105 ^b^
ALT activity, U/L, mean ± SD	59.4 ± 24.0	46.1 ± 22.0
Liver biopsy, mean ± SD		
Grading	1.11 ± 0.49	0.92 ± 0.46
Staging	0.68 ± 0.49	0.63 ± 0.53
Treatment-naïve, No. (%)	101 (85)	26 (51)
Retherapy, No. (%)	18 (15)	25 (49)

^a^ Abbreviations: IFn; Interferon, RBV; Ribavirin

^b^ In 3 cases, HCV RnA was assessed only qualitatively.

All the children had to undergo liver biopsy and liver untrasonography as a prerequisite for enrollment and treatment. Liver biopsy specimens were scored according to the modified Scheuer scale and were assigned a grade between 0 and 4 for necroinflammation and a stage between 0 and 4 for fibrosis [9]. The severity of liver disease did not exceed grade 2 and stage 2 in any child. Patients with chronic liver disease other than CHC or those who were co-infected with hepatitis B virus or human immunodeficiency virus were not considered eligible. The children were divided into 2 groups-the first group (n = 119) of children were administered a 48-week course of recombinant IFN-α-2b (Intron A) subcutaneously at a dosage of 3 MU 3 times a week and RBV at a dosage of 15 mg/kg per day orally, and the second group (n = 51) of children were administered peg-IFN-α-2b (PegIntron) at a dosage of 1.5 μg/kg per week subcutaneously and RBV 15 mg/kg per day orally for 48 weeks. The dose of IFN was not adjusted but that of RBV was in the case of 2 children from group II. Hematological growth factors and erythropoietin were not used. SVR was defined as undetectable HCV RNA in the serum 24 weeks after the end of treatment.

Serum HCV-RNA viral load was determined at the baseline, at the 12th and 48th weeks during treatment, and after 24 weeks of untreated follow-up (study week 72) by performing quantitative polymerase chain reaction (PCR) (COBAS® AmpliPrep/COBAS TaqMan® HCV Test; Roche Diagnostics Geneva, Switzerland), which had a detection limit of 43 IU/mL. The primary efficacy variable was the proportion of patients who attained SVR. The genotypes of HCV were identified by performing the INNOGENETICS INNO-LiPA HCV II test. Hematological parameters (hemoglobin levels, leukocyte counts, and platelet counts) were evaluated at baseline, at the 12th and 24th weeks, and at the end of the therapy by using Sysmex 4500.

### 3.1. Statistical Analysis

Hemoglobin levels, leukocyte counts, and platelet counts were analyzed by using descriptive statistics. Means and standard deviations were calculated for values recorded at baseline, at the 12th and 24th weeks, and at the end of the treatment. We used Student's t test, Mann-Whitney U test, and the chi-squared test for the statistical analyses.

## 4. Results

**Figure 1:**
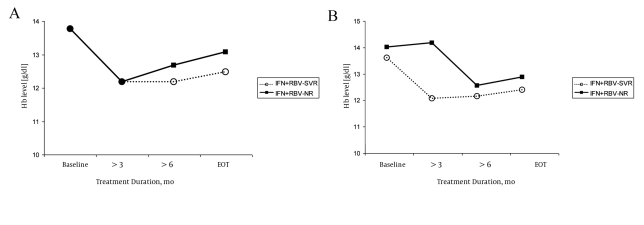
Hemoglobin Levels observed in Children During Treatment. Group I (A) and group II (B)

**Figure 2:**
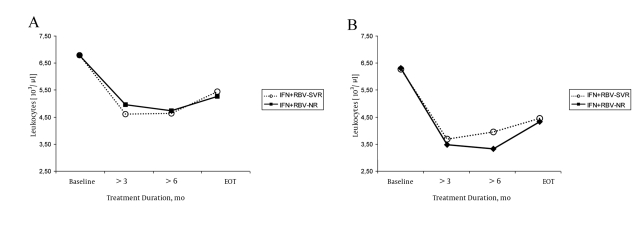
Leukocyte Count observed in Children During Treatment. Group I (A) and group II (B)

**Figure 3:**
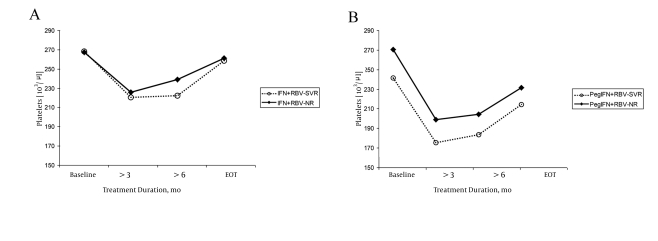
platelets Count observed in Children During Treatment. Group I (A) and Group II (B)

SVR was achieved in 86 (50%) of the 170 treated children: 62 (51%) of 119 children from group I and 24 (47%) of 51 from group II. In the baseline examination of both the groups of patients, no statistically significant differences were observed in the serum hemoglobin levels, leukocyte count, and platelet count between the children who achieved SVR (responders) and those who did not (non-responders). In both the treatment groups, we observed significant and similar decrease in the hematological parameters that were analyzed during the therapy. The serum hemoglobin levels decreased during treatment in the responders and non-responders from both the groups (Figure 1 A and 1B). In group I, the mean serum hemoglobin levels after 24 weeks of the treatment and at the end of the therapy were significantly lower in children who achieved SVR, especially treatment-naïve children (P < 0.05) (Table 2). In group II, the mean serum hemoglobin levels were significantly lower in the responders after 12 weeks of therapy. During the later course of the treatment, the differences were not statistically significant. In the responders and non-responders from both the groups, the leukocyte counts decreased during the treatment, and after the 12th week, they were significantly lower than the baseline measurement. The decrease was more marked in children treated with peg-IFN-α-2b and RBV (PegIFN + RBV) (Figure 2AandFigure 2B). The leukocyte counts after 12 weeks were significantly lower in children treated with Peg-IFN-α-2b + RBV than in those treated with IFN and RBV (SVR, P < 0.05 and non-responders NR, P < 0.05) (Table 3).The platelet counts during the therapy were within the normal range; they decreased during the treatment and were significantly lower after 12 weeks than at the baseline in both the responders and non-responders from both the groups (P < 0.05) (Figure 3A andFigure 3B). After 12 weeks of treatment, the platelet count was lower in children from group II who had achieved SVR (Table 4).

**Table 2:** Serum Hemoglobin Levels (g/dL) in Treated Children

	**Treated children, No. **	**Baseline**	**After 12 Wk**	**After 24 Wk**	**End of Treatment**
		**Group I; IFN + RBV (n = 119)**			
SVR a	62	13.7 ± 1.2	12.1 ± 1.1^b^	12.1 ± 0.9 c	12.4 ± 1.2 c
Treatment-naïve	53	13.7 ± 1.3	12.2 ± 1.1	12.1 ± 0.9 c	12.6± 1.2
Retherapy	9	13.3 ± 0.8	11.5 ± 1.3	11.6 ± 0.8	11.6 ± 1.1
NR a	57	13.7 ± 1.1	12.1 ± 1.3 a	12.6±1.4 c	13.0±1.2 c
Treatment-naïve	48	13.7 ± 0.9	12.2 ± 1.2	12.6 ± 1.1 c	13.0 ± 1.1
Retherapy	9	13.8 ± 1.6	11.9 ± 1.4	12.2 ± 1.2	12.4 ± 1.5
		**Group II; Treated With Peg-IFN-α-2b + RBV (n = 51)**			
SVR	24	13.5 ± 1.2 b	11.6 ± 1.1 b, c	11.7 ± 1.1	12.0 ± 1.5
Treatment-naïve	15	13.7 ± 1.3	11.5 ± 1.2	11.8 ± 1.0	12.0 ± 1.6
Retherapy	9	13.2 ± 1.3	11.8 ± 0.9	11.4 ± 1.1	12.0 ± 1.4
NR	27	14.0 ± 1.0 b	12.4 ± 1.6 b, c	12.2 ± 1.5	12.6 ± 1.7
Treatment-naïve	10	13.7 ± 1.1	12.0 ± 1.2	12.6 ± 2.1	12.1 ± 1.4
Retherapy	17	14.2 ± 0.9	12.7 ± 1.8	12.0 ± 1.1	12.9 ± 1.8

^a^ Abbreviations: nR; non-responders, SVR; Sustained virological response

^b^ Statistically significant differences, P < 0.05 (time dependent in one group)

^c^ Statistically significant differences, P < 0.05 (between groups I and II)

**Table 3:** Leukocyte Count (103/μL) in Treated Children

	**Treated children, No. **	**Baseline**	**After 12 Wk**	**After 24 Wk**	**End of Treatment**
		**Group I; IFN + RBV (n = 119)**			
SVR a	62	13.7 ± 1.2	12.1 ± 1.1^b^	12.1 ± 0.9 c	12.4 ± 1.2 c
Treatment-naïve	53	13.7 ± 1.3	12.2 ± 1.1	12.1 ± 0.9 c	12.6± 1.2
Retherapy	9	13.3 ± 0.8	11.5 ± 1.3	11.6 ± 0.8	11.6 ± 1.1
NR a	57	13.7 ± 1.1	12.1 ± 1.3 a	12.6±1.4 c	13.0±1.2 c
Treatment-naïve	48	13.7 ± 0.9	12.2 ± 1.2	12.6 ± 1.1 c	13.0 ± 1.1
Retherapy	9	13.8 ± 1.6	11.9 ± 1.4	12.2 ± 1.2	12.4 ± 1.5
		**group II; Treated With Peg-IFN-α-2b + RBV (n = 51)**			
SVR	24	13.5 ± 1.2 b	11.6 ± 1.1 b, c	11.7 ± 1.1	12.0 ± 1.5
Treatment-naïve	15	13.7 ± 1.3	11.5 ± 1.2	11.8 ± 1.0	12.0 ± 1.6
Retherapy	9	13.2 ± 1.3	11.8 ± 0.9	11.4 ± 1.1	12.0 ± 1.4
NR	27	14.0 ± 1.0 b	12.4 ± 1.6 b, c	12.2 ± 1.5	12.6 ± 1.7
Treatment-naïve	10	13.7 ± 1.1	12.0 ± 1.2	12.6 ± 2.1	12.1 ± 1.4
Retherapy	17	14.2 ± 0.9	12.7 ± 1.8	12.0 ± 1.1	12.9 ± 1.8

^a^ Abbreviations: nR; non-responders, SVR; Sustained virological response

^b^ Statistically significant difference, P < 0.05 (time dependent in one group)

^c^ Statistically significant difference, P < 0.05 (between groups 1 and 2)

**Table 4:** Platelet Count (103/μL) in treated children

	**Treated children, No.**	**Baseline**	**After 12 Wk**	**After 24 Wk**	**End of Treatment**
		**IFN + RBV (n = 119)**					
SVR a	62	268 ± 66 b	221 ± 57 b	236 ± 66	261 ± 78
Treatment-naïve	53	267 ± 62	220 ± 57	235 ± 66	255 ± 79
Retherapy	9	273 ± 86	229 ± 61	243 ± 66	289 ± 71
NR a	57	269 ± 77 b	215±62 b	217 ± 61 b	258 ± 86
Treatment-naïve	48	272 ± 76	217 ± 61	221 ± 61	261 ± 90
Retherapy	9	254 ± 80	205 ± 66	203 ± 64	229 ± 50
		**Peg-IFN-α-2b + RBV (n = 51)**			
SVR	24	245 ± 65 b	172 ± 45 b	181 ± 55	215 ± 64
Treatment-naïve	15	244 ± 72	166 ± 46	171 ± 60	190 ± 50
Retherapy	9	246 ± 55	182 ± 45	196 ± 44	247 ± 69
NR	27	277 ± 70 b	198±45 b	204 ± 50	234 ± 45
Treatment-naïve	10	247 ± 48	182 ± 47	194 ± 49	215 ± 37
Retherapy	17	295 ± 77	208 ± 42	209 ± 51	244 ± 46

^a^ Abbreviations: nR; non-responders, SVR; Sustained virological response

^b^ Statistically significant difference, P < 0.05 (time dependent in one group)

## 5. Discussion

Hematological complications such as anemia, thrombocytopenia, and leucopenia are common and frequent side effects of combination therapy with interferon and RBV, but their intensity ranges from mild to moderate. Such hematological complications were observed in our study as well. Such complications need to be monitored during the therapy [10][11][12][13]. Hematologic toxicity is one of the most common reasons for dose reduction or discontinuation of the treatment in patients with CHC infection. Dose reduction or treatment discontinuation may lower the SVR and result in 10-20% reduction in the patients' adherence to the treatment regimen [14]. The higher rate of cytopenia in adults, mainly in the elderly, can be explained by their lower level of baseline hemoglobin and neutrophil and platelet counts [15]. Some authors have suggested that severe cytopenia is a marker for increased tumor-necrosis-factor (TNF) activity in patients, and this increased TNF activity translates into higher SVR [16]. It is known that proinflammatory cytokines such as TNF-α and IFN-γ induce cell adhesion in endothelial cells and promote transmigration of leukocytes across endothelial cells. Ozaki et al. showed that combination therapy with TNF-α and IFN-γ causes disappearance of junctional adhesion molecules (JAMs) from intercellular junctions, and JAMs change their distribution in response to proinflammatory cytokines. This redistribution of JAM might be associated with the decrease in transendothelial migration of leukocytes at inflammatory sites [17].

It is unclear if the decrease in the leukocyte count in patients treated with IFN-α is only a side effect or a characteristic of IFN action and a marker of beneficial prognosis. In a previous study, we analyzed the dynamics of leukocyte count associated with the end-of-treatment response (ETR). All the examined children who achieved ETR, initially had shown early virological response - EVR, ALT normalization in the 12th week of therapy, and decrease in the leukocyte count. In the non-responders, the decrease in the leukocyte count was lower than that in the first group (a decrease of 23% and 37%, respectively). Decreased values of the leukocyte count did not warrant a modification in the treatment dose or schedule. Our study proves that a mild decrease in the leukocyte count in the 12th week of therapy is associated with EVR and ALT-activity normalization in the 12th week, during ETR, and in the follow-up period [18].

In a study by Suwantarat, at the end of the treatment, leukocyte and platelet counts were significantly lower in patients who had achieved SVR than in those who had not. Authors suggested that patients who developed leucopenia or thrombocytopenia during the IFN therapy responded well to the therapy, and the side effects, if not severe, may not be indications for withholding or reducing the dose of IFN [16]. In a study by Sievert et al, a higher SVR was achieved by patients who were infected with genotype 1 HCV and who developed anemia or decreased hemoglobin levels when treated with Peg-IFN-α-2b + RBV [19]. Similar observations were made in our study. The hemoglobin levels and leukocyte and platelet counts during the treatment, especially after 12 weeks of therapy, were lower in children who achieved SVR than in those who did not. In both the groups, a statistically significant decrease was observed in the mean serum hemoglobin levels in children who had attained SVR. A more statistically significant decrease was observed in the serum hemoglobin levels after 12 weeks of therapy with Peg-IFN-α-2b + RBV in children who had achieved SVR than in the non-responders (P < 0.05). Analysis of leukocyte count according to the type of IFN administered showed that peg-IFN-α-2b had a more marked influence on the decrease in leukocyte count than recombinant IFN did. We observed a more statistically significant decrease in the leukocyte count after 12th and 24th weeks of treatment and at the end of therapy in children treated with peg-IFN-α-2b + RBV than in those treated with recombinant IFN + RBV (SVR, P < 0.05).

Similar results were obtained in the case of platelet count in children treated with peg-IFN-α-2b and RBV. In this group, we observed a decrease in the platelet count after 3 months of treatment, and this decrease was higher in the responders than in the non-responders. A stronger action of peg-IFN-α-2b associated with a more a marked decrease in the leukocyte and platelet count and a higher SVR induction was observed in the children treated with peg-IFN-α-2b and RBV; this may be explained by the slightly higher treatment response in this group than that observed in the group treated with recombinant IFN despite the high ratio of children undergoing retherapy in children treated with peg-IFN-α-2b and RBV. The SVR of the naïve and retreated children was 53% and 50%, respectively, in group I and 77% and 37%, respectively, in group II. Among 24 patients who achieved SVR during treatment with peg-IFN-α-2b and RBV, 15 were naïve responders and 9 were previously treated patients. The highest decrease in the leukocyte and platelet counts were observed in treatment-naïve children treated with peg-IFNα-2b and RBV. However, it is important to note that 37% of the previously treated children in this group attained SVR. Other factors that influenced SVR in this group of patients were the higher mean baseline HCV viral load and the lower ALT activity than that observed in children from group I. Our data have shown that a mild decrease in hemoglobin levels and leukocyte and platelet counts during treatment with IFN and RBV in children with CHC infection may be factors associated with SVR induction. Thus, close monitoring of different hematological parameters during treatment is necessary.
